# Schnittbilddiagnostik im InEK‑Benchmark: Eine Chance für die Radiologie

**DOI:** 10.1007/s00117-021-00963-8

**Published:** 2022-01-14

**Authors:** V. Wienicke, T. Denecke, J. Henkelmann, R. Jacob, Nikolaus von Dercks

**Affiliations:** 1 Stabsstelle Medizincontrolling, Universitätsklinikum Leipzig, Liebigstr. 18, 04103 Leipzig, Deutschland; 2 Klinik und Poliklinik für diagnostische und interventionelle Radiologie, Universitätsklinikum Leipzig, Leipzig, Deutschland; 3 Vorstand, Universitätsklinikum Leipzig, Leipzig, Deutschland

**Keywords:** Schnittbildgebung, DRG, Klinische Prozesse, Magnetresonanztomographie, Computertomographie, Cross-sectional diagnostic imaging, DRG, Clinical processes, Magnetic resonance imaging, Computed tomography

## Abstract

**Hintergrund:**

Anhand der vom Institut für das Entgeltsystem im Krankenhaus (InEK) kalkulierten Fallpauschalen ist ein Vergleich der eigenen Leistungsdaten gegenüber allen nach der DRG („diagnosis-related groups“, diagnosebezogene Gruppen) abrechnenden Kliniken in Deutschland möglich. Ziel der vorliegenden Arbeit ist es, Über- oder Unterschreitungen von CT- oder MRT-Untersuchungen im Vergleich mit den InEK-Daten zu ermitteln und mögliche Verbesserungspotenziale zu erschließen.

**Methodik:**

Die InEK-Kalkulationsdaten für 2021 wurden zur Bildung von Vergleichskennzahlen der CT- und MRT-Diagnostik auf DRG-Ebene herangezogen. Auf Fallebene wurden Daten eines universitären Maximalversorgers auf Gesamthaus‑, Klinik‑, DRG- und Hauptdiagnosen-Ebene gegenübergestellt.

**Ergebnis:**

Auf Gesamthausebene zeigt sich eine Überschreitung der MRTs um 1025 und der CTs um 371 gegenüber InEK. Die Analyse nach Fachabteilungen ergab am Beispiel der Neurologie eine Überschreibung der MRTs gegenüber InEK um 489 sowie eine Unterschreitung der CTs um $-$620. Der Benchmark der DRGs zeigte in beiden Untersuchungsmodalitäten insbesondere die DRG B70B als Treiber der Abweichungen (MRT + 42,7; CT − 273). Die identifizierten Abweichungen lassen sich auf Hauptdiagnosen-Ebene weiter herunterbrechen.

**Diskussion:**

Das Bewusstsein über eine überdurchschnittliche Schnittbilddiagnostik kann einen wichtigen Anstoß zur Weiterentwicklung der Behandlungspfade einer Klinik bilden. Die Methodik des InEK-Benchmarks ist für jedes Krankenhaus anwendbar und identifiziert valide bereits erbrachte Leistungen und Prozesse mit einem Verbesserungspotenzial. Die Prüfung beeinflussender Faktoren sowie die Bewertung durch Mediziner und Kaufleute bildet die Voraussetzung für Akzeptanz und Erfolg der daraus generierten Maßnahmen.

## Abstract (en)

**Background:**

Based on the lump sum system which is calculated by the InEK (Institut für das Entgeltsystem im Krankenhaus), it is possible to compare one hospital’s own performance data with all hospitals in Germany billing according to the Diagnosis-Related Group (DRG). The aim of this article is to investigate exceeding or undercutting of computed tomography (CT) or magnetic resonance imaging (MRI) in comparison to the calculations made by the InEK to reveal potential for improvement.

**Methods:**

InEK calculation data for 2021 were used to form comparative ratios for CT and MRI diagnostics at the DRG level. Data from a university hospital was compared at the hospital, departments, DRG and main diagnosis levels.

**Results:**

At the hospital level, there were 1025 more MRIs and 371 more CTs compared to InEK. Analysis by department showed, e.g., in neurology, 489 more MRIs and 620 fewer CTs. The benchmark of the DRGs showed in particular that DRG B70B was the driver of deviations in both diagnostic modalities (MRI $+42.7;$ CT $-273$). The identified deviations can be further analysed at the main diagnosis level.

**Conclusion:**

Awareness of above-average use of cross-sectional imaging diagnostics can provide impetus for further development of clinical pathways of a hospital. The methodology of the InEK benchmark is applicable to every hospital and is able to identify processes with potential for improvement. The review of influencing factors as well as the evaluation by clinicians and economists are prerequisite for acceptance and success of the measures generated from the benchmark.

Die Modernisierung der Medizin geht aufgrund des steigenden Qualitäts- und Sicherheitsanspruches mit einer vermehrten Inanspruchnahme radiologischer Leistungen einher.

Den steigenden Kosten für die radiologische Versorgung steht die limitierte Finanzierung der stationären Krankenhausbehandlung im DRG-System entgegen. Eine Orientierung an anderen Krankenhäusern kann beim Vergleich im Rahmen eines Benchmarks dabei helfen, eine erhöhte Inanspruchnahme radiologischer Leistungen zu identifizieren und so Strategien zum effizienten Ressourceneinsatz zu entwickeln.

Dieser Beitrag beschreibt die Methodik eines Benchmarks mit den InEK-Kalkulationsdaten zur Identifikation von Einsparungspotenzial in der Schnittbilddiagnostik.

## Hintergrund

Die duale Krankenhausfinanzierung in Deutschland steht seit Jahren in der Kritik. Sowohl die Pauschalierung der Betriebskosten in Form der Fallpauschalen als auch die unzureichende Investitionsfinanzierung der Länder lassen den ökonomischen Druck auf Leistungserbringer wachsen, weswegen das Controlling auch im Hinblick auf kostenintensive Elemente der Krankenversorgung, wie z. B. der Radiologie, von großer Bedeutung ist. Als Grundlage vieler Therapieentscheidungen sind radiologische Leistungen ein essenzieller Bestandteil in der klinischen Diagnostik und regelhaft als standardisierte Behandlungspfade in Leitlinien für viele Diagnosen vorgeschrieben. Die stetige Individualisierung und Modernisierung von Therapien und Möglichkeiten der diagnostischen Bildgebung sowie der hohe Qualitäts- und Sicherheitsanspruch von Arzt und Patient führen dazu, dass radiologische Leistungen häufiger und multimodal in Anspruch genommen werden. Ein überhöhtes Anforderungsverhalten in der Computertomographie (CT) und Magnetresonanztomographie (MRT) in der Klinik kann durch Knappheit der Kapazitäten (Geräte bzw. Zeit und Personal der Radiologie sowie Infrastruktur) zu wachsenden Wartezeiten und steigenden Verweildauern (VWD) der behandelnden Fachabteilungen führen. Probleme mit Abrechnungsprüfungen durch den Medizinischen Dienst sowie sinkende Fallzahlen wegen mangelnder Bettenkapazität sind die Folge. In dem auf kurze Verweildauern und einen steigenden Wettbewerb ausgerichteten Vergütungssystem der „diagnosis-related groups“ (diagnosebezogene Gruppen, DRG) wird eine überproportionale Diagnostik nicht ausreichend refinanziert [1]. Eine Steuerung der kostenintensiven CT- und MRT-Untersuchungen ist für einzelne Fachbereiche über die standardisierten Klinikberichte mit Kennziffern wie der VWD oder dem Case-Mix-Index jedoch nicht möglich.

Ein fachübergreifendes Monitoring-Instrument des Controllings ist der Benchmark. Anhand ausgewählter Kennzahlen wird dabei im kontinuierlichen Vergleich der „Best-Practice-Ansatz“ identifiziert. Der Vergleich mit Wettbewerbern auf dem klinischen Gebiet kann somit Ansatzpunkte zur besseren Versorgung liefern [2]. Neben dem Erkenntnisgewinn bieten Benchmark-Ergebnisse eine objektive Datengrundlage, welche den sachlichen Diskurs über die Ursachen des Anforderungsverhaltens zwischen dem Bereich des Controllings und der klinischen Leistungserbringer fördern kann [8].

Die DRG-Kalkulationsdaten des Instituts für das Entgeltsystem im Krankenhaus (InEK) bieten eine hohe Transparenz in der stationären Gesundheitsversorgung. Sie basieren für das Jahr 2021 auf den Leistungsdaten (§ 21-Datensatz) aller nach DRG abrechnenden Kliniken sowie den Kostendaten der Kalkulationskrankenhäuser aus dem Jahr 2019. Zahlreiche Publikationen stellen bereits den Mehrwert des InEK-Benchmarks auf Basis der Kostendaten vor [3, 7, 8]. Dabei werden jedoch auch Limitationen in der Aussagekraft benannt. Unterschiede in der Kosten- und Erlösverteilung, der hohe Aggregationsgrad der Fallpauschalen [8] sowie die Abhängigkeit der nach InEK-Kalkulation getrennten Kostenstellen [3] führen innerhalb des Kosten-Benchmarks zu Verwerfungen. Trotz der genannten Einschränkungen stellt der InEK-Benchmark eine äußerst potente Datenbasis dar, die in Verbindung mit weiterführenden Analysen Zusammenhänge der Kosten- und Leistungsstruktur offenlegen können [3, 8].

Die folgende Untersuchung vergleicht auf Ebene einzelner Fallpauschalen die Inanspruchnahme der Schnittbilddiagnostik an einem universitären Maximalversorger mit den DRG-Kalkulationsdaten des InEK.

## Methodik

**Figure 1:**
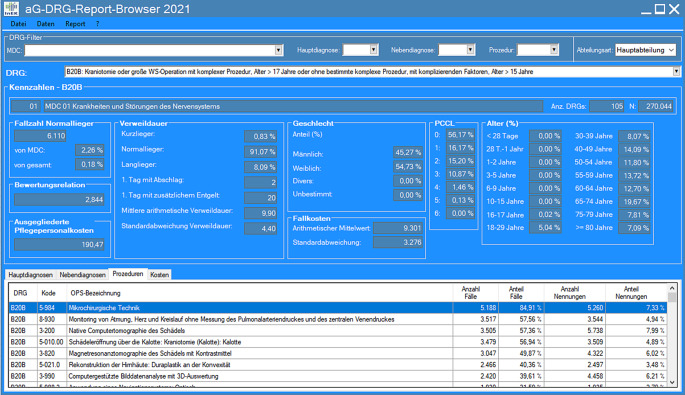

Die Grundlage des Vergleichs bilden die Fallpauschalen mit den quantifizierten CT- und MRT-Prozeduren. Hierzu betrachteten wir alle stationären Behandlungsfälle des Universitätsklinikums Leipzig (UKL) 2019. Als Vergleichsgruppe für den Benchmark wurden die jährlich veröffentlichten Kalkulationsdaten des InEK in Form des aG-DRG-Report-Browsers herangezogen (Abb. 1). Die Aufbereitung und Auswertung der Daten erfolgte mittels Microsoft Excel in der Version 2016 (Microsoft Corporation, Redmond, WA, USA).

Datengrundlage des Report-Browsers sind die Abrechnungsdaten aller deutschen Krankenhäuser von 2019. Die Dateien „Kopfdaten“ und „Prozeduren“ sind die Grundlage des aG-DRG-Report-Browser und bieten die Basis bei der Bildung der Vergleichskennzahlen. Diese enthalten die DRGs mit der dazugehörigen Anzahl an Fällen sowie die Prozedurenkodes mit der Anzahl der Nennung je DRG. Wir kumulierten die CT- und MRT-Kodes (3–2* und 3–8*) nach Fallpauschalen, um die Summe der kalkulierten CT und MRT je DRG zu ermitteln. Die Division der Anzahl der Nennung durch die Fallanzahl ergab anschließend die durchschnittlichen Quoten der CT und MRT je DRG.

$${CT-\text{Quote}}_{\mathrm{DRG}}^{\mathrm{InEK}}=\frac{{\sum }_{\text{Prozeduren}\,3-2*\,\text{einer}\,\mathrm{DRG}}^{\mathrm{InEK}}}{{\sum }_{\text{F\"alle}\,\text{einer}\,\mathrm{DRG}}^{\mathrm{InEK}}}$$

$${\mathrm{MRT}-\text{Quote}}_{\mathrm{DRG}}^{\mathrm{InEK}}=\frac{{\sum }_{\text{Prozeduren}\,3-8*\,\text{einer}\,\mathrm{DRG}}^{\mathrm{InEK}}}{{\sum }_{\text{F\"alle}\,\text{einer}\,\mathrm{DRG}}^{\mathrm{InEK}}}$$

Im Jahr 2019 wurden im UKL 56.917 Patienten stationär behandelt. Die Kodierung dieser Fälle bildet die mit dem InEK-Benchmark zu vergleichende Gruppe. Die analog der InEK-Kalkulationsdaten erfassten Fälle aus dem Jahr 2019 gruppierten wir in einer Simulation (Übergangsgrouping) mit dem Programm ID EFIX (ID Berlin GmbH & Co. KGaA) nach den Kodierregelungen für 2021. Die Fallübersicht des UKL enthielt daraufhin die simulierten DRGs, Fallnummern, Hauptdiagnosen (HD) in Form des 4‑ bzw. 3‑Stellers, entlassene Fachabteilung sowie die Anzahl der erbrachten CT und MRT. Anhand der simulierten DRGs ordneten wir die ${CT-\text{Quote}}_{\mathrm{Fall}}^{\mathrm{InEK}}$ sowie die ${\mathrm{MRT}-\text{Quote}}_{\mathrm{Fall}}^{\mathrm{InEK}}$den Behandlungsfällen zu und bildeten das Delta (∆) aus den UKL- und InEK-Werten je Fall (${\Delta CT-\text{Quote}}_{\mathrm{UKL}}^{\mathrm{InEK}}$ bzw. ${\sum \Delta \mathrm{MRT}-\text{Quote}}_{\mathrm{UKL}}^{\mathrm{InEK}}$). Die Summierung des ∆ beider Untersuchungsmodalitäten ergab das ${\Updelta \text{Schnittbild}}_{\mathrm{Fall}}^{\mathrm{UKL}}$. Die erschlossene Über- oder Unterschreitung auf Fallebene bildet die Basis der Betrachtungen auf Klinik- (${\Delta CT}_{\mathrm{entl}.\text{Klinik}}^{\mathrm{UKL}}$ bzw. ${\Delta \mathrm{MRT}}_{\mathrm{entl}.\text{Klinik}}^{\mathrm{UKL}}$) und UKL-Ebene ($\Updelta CT_{\mathrm{UKL}}$ bzw. $\Updelta \mathrm{MRT}_{\mathrm{UKL}}$). Nachfolgend ist die Berechnung auf Ebene des Gesamthauses (UKL) aufgeführt:

$$\Updelta CT_{\mathrm{UKL}}=\sum CT_{\mathrm{UKL}}-{\sum \Delta CT-\text{Quote}}_{\mathrm{UKL}}^{\mathrm{InEK}}$$

$$\Updelta \mathrm{MRT}_{\mathrm{UKL}}=\sum \mathrm{MRT}_{\mathrm{UKL}}-{\sum \Delta \mathrm{MRT}-\text{Quote}}_{\mathrm{UKL}}^{\mathrm{InEK}}$$

$$\Updelta \text{Schnittbild}_{\mathrm{UKL}}=\Updelta CT_{\mathrm{UKL}}+\Delta \mathrm{MRT}_{\mathrm{UKL}}$$

Wir ermittelten zunächst das $\Updelta$ auf UKL-Ebene für alle Fälle, um anschließend auffällige Fachbereiche nach DRGs und Hauptdiagnosen weiterführend zu analysieren. Die Betrachtung anhand von absoluten Werten identifizierte Kliniken und Leistungen mit hoher Abweichung und hoher Fallzahl. Die Analyse anhand von relativen Werten (Quoten) ermöglichte indes die Bestimmung von Abteilungen, DRG und Hauptdiagnose (HD) mit geringer Fallzahl bei hoher Überschreitung. Im Sinne des Benchmarks berechneten wir die CT- und MRT-Quoten gleichsam für das InEK. Nachfolgend ist die Formel beispielhaft für die Fachabteilungsebene der UKL-Leistungsdaten aufgeführt.

$${CT-\text{Quote}}_{\mathrm{entl}.\text{Klinik}}^{\mathrm{UKL}}=\frac{{\sum CT}_{\mathrm{entl}.\text{Klinik}}^{\mathrm{UKL}}}{{\sum }_{\text{\"alle}\,\mathrm{entl}.\text{Klinik}}^{\mathrm{UKL}}}$$

$${\mathrm{MRT}-\text{Quote}}_{\mathrm{entl}.\text{Klinik}}^{\mathrm{UKL}}=\frac{{\sum \mathrm{MRT}}_{\mathrm{entl}.\text{Klinik}}^{\mathrm{UKL}}}{{\sum }_{\text{F\"alle}\,\mathrm{entl}.\text{Klinik}}^{\mathrm{UKL}}}$$

Zur Verdeutlichung der Methodik des InEK-Benchmarks mit Leistungsdaten sollen im Folgenden ausgewählte Ergebnisse vorgestellt werden. Diese enthalten die Ebene des Universitätsklinikums Leipzig (UKL) sowie exemplarisch die Kennzahlen der Klinik für Neurologie (${CT-\text{Quote}}_{\text{Neuro}}^{\mathrm{UKL}}$und ${\mathrm{MRT}-\text{Quote}}_{\text{Neuro}}^{\mathrm{UKL}}$) mit jeweils einer für diesen Fachbereich ausschlaggebenden DRG und Hauptdiagnose.

## Ergebnisse

### Gesamthaus UKL

Das UKL überschreitet im Benchmark die Schnittbilddiagnostik des InEK im $\Updelta \mathrm{MRT}_{\mathrm{UKL}}$ um $+1025$ sowie im $\Updelta CT_{\mathrm{UKL}}$ um $+371$. Unter Berücksichtigung der Fallzahl ergibt sich eine $\mathrm{MRT}-\text{Quote}_{\mathrm{UKL}}$ von $15\%$ sowie eine $\mathrm{MRT}-\text{Quote}_{\mathrm{InEK}}$ von $13,1\%$ und eine $CT-\text{Quote}_{\mathrm{UKL}}$ von $48,5\%$ sowie eine $CT-\text{Quote}_{\mathrm{InEK}}$ von $47,8\%$.

### Kliniken und Abteilungen

**Figure 2:**
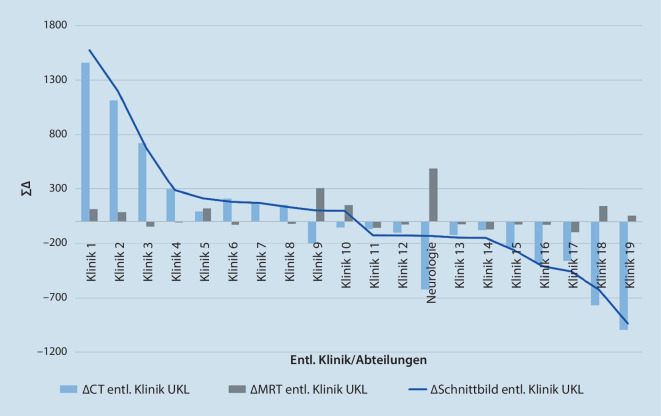

Die Ebene der Kliniken und Abteilungen eröffnet einen Einblick auf die Zusammensetzung der kumulierten Benchmarkergebnisse des Gesamthauses (Abb. 2). Die 20 Kliniken mit dem höchsten ${\mathit{\Delta }\text{Schnittbild}}_{\mathrm{entl}.\text{Klinik}}^{\mathrm{UKL}}$ fallen durch Überschreitung als auch durch Unterschreitung auf. Die Spreizung des ${\Delta CT}_{\mathrm{entl}.\text{Klinik}}^{\mathrm{UKL}}$ verläuft zwischen Klinik 1 mit einem Δ von $+1575$ bis hin zur Klinik 19 mit $-938$ im ${\Delta CT}_{\mathrm{entl}.\text{Klinik}}^{\mathrm{UKL}}$. Das ${\Delta \mathrm{MRT}}_{\mathrm{entl}.\text{Klinik}}^{\mathrm{UKL}}$ wird insbesondere von der Klinik für Neurologie mit $+489$ über- und von Klinik 17 unterschritten ($-97$). Nachfolgend benennen wir zur Verdeutlichung der Analysetiefe des InEK-Benchmarks die Ergebnisse der Klinik für Neurologie auf den jeweiligen Analyseebenen.

Anhand des Leistungsbenchmarks konnten wir für die Klinik für Neurologie ein ${\Delta \mathrm{MRT}}_{\text{Neuro}}^{\mathrm{UKL}}$ von $+489$ $({\sum \mathrm{MRT}}_{\text{Neuro}}^{\mathrm{UKL}}=\ 2224)$ ermitteln. Während das InEK bei rund 7 von 10 Patienten (${\mathrm{MRT}-\text{Quote}}_{\text{Neuro}}^{\mathrm{InEK}}=\ 71,7\mathrm{\% }$) die genannte Diagnostik kalkulierte, kam die Magnetresonanztomographie bei 9 von 10 Patienten (${\mathrm{MRT}-\text{Quote}}_{\text{Neuro}}^{\mathrm{UKL}}=91,8\mathrm{\% }$) der Neurologie am UKL zum Einsatz. Gleichzeitig unterschritt die Klinik die ${\sum CT}_{\text{Neuro}}^{\mathrm{InEK}}$ um $-620$ Computertomographien (${\sum CT}_{\text{Neuro}}^{\mathrm{UKL}}=\ 2878$). Es hat den Anschein, dass bei bestimmten Fallkonstellationen eine unmittelbare MRT-Untersuchung der CT vorgezogen wird. Dies kann auf Ebene der DRGs und Hauptdiagnosen weiter analysiert werden.

### DRG-Fallpauschalen

**Figure 3:**
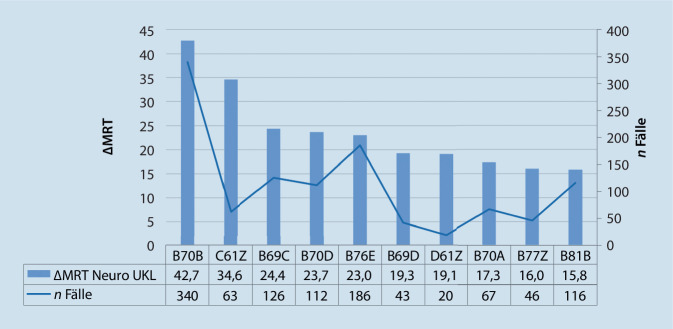

Die in der Methodik vorgestellte Analyse auf Ebene der Fallpauschalen erfolgte für die Top-10-DRGs einer Fachabteilung, getrennt nach Untersuchungsmodalität (Abb. 3). Hierdurch ist die Darstellung der Abweichungen des Fachbereichs auf die wesentlichen Fallpauschalen möglich.

Im Benchmark der MRT-Untersuchungen dominiert die DRG B70B „Apoplexie mit neurologischer Komplexbehandlung des akuten Schlaganfalls […]“ in der Analyse mit $+42,7{\Delta \mathrm{MRT}}_{\text{Neuro}}^{\mathrm{UKL}}$ gefolgt von den DRGs C61Z ($+34,6{\Updelta \mathrm{MRT}}_{\text{Neuro}}^{\mathrm{UKL}}$), B69C ($+24,4{\Updelta \mathrm{MRT}}_{\text{Neuro}}^{\mathrm{UKL}}$), B70D ($+23,7{\Updelta \mathrm{MRT}}_{\text{Neuro}}^{\mathrm{UKL}})$, B76E ($+23,0{\Updelta \mathrm{MRT}}_{\text{Neuro}}^{\mathrm{UKL}})$ sowie weiteren DRGs mit einem ${\Delta \mathrm{MRT}}_{\text{Neuro}}^{\mathrm{UKL}}< 20$.

Die Ergebnisse zeigen, dass die Fälle der DRG B70B zudem das höchste $$\Updelta$$ der CT-Leistungen mit einem negativen $$\Updelta$$ von $$-273$$ aufweisen. Ein $${\Updelta \mathrm{C}T}_{\text{Neuro}}^{\mathrm{UKL}}$$ von $$< -20$$ weisen zudem die DRGs B70D ($$-93,6)$$, B70A ($$-71,5)$$ sowie B69C ($$-59,3)$$ auf.

### Hauptdiagnosen

**Figure 4:**
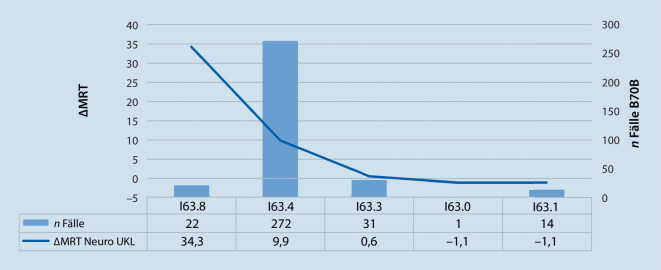

Der Benchmark auf HD-Ebene wird für die DRG B70B (Abb. 4) vorgestellt. Auch wenn die Berechnungen des InEK für die Untersuchungsmodalitäten auf DRG-Basis erfolgen, kann auf Fallebene für das UKL jeder HD innerhalb der DRG ein ${\Updelta \mathrm{MRT}}_{\text{Neuro}}^{\mathrm{UKL}}$ sowie eine ${\Updelta CT}_{\text{Neuro}}^{\mathrm{UKL}}$ gegenübergestellt werden.

Die HD I63.8 „Sonstiger Hirninfarkt“ führte bei 22 Fällen mit Zuordnung zur Fallpauschale B70B zu einem ${\Delta \mathrm{MRT}}_{\text{Neuro}}^{\mathrm{UKL}}$ von $+34,3$. Patienten mit dieser HD und DRG erhalten folglich in der Klinik für Neurologie mehr MRT-Diagnostik, als dies die Kalkulationsdaten des InEK ausweisen.

In der Betrachtung der CT unterschritt die Neurologie die InEK-Werte in der DRG B70B. Entscheidend für die Abweichung ist die HD I63.4 mit einem ${\Delta CT}_{\text{Neuro}}^{\mathrm{UKL}}$von $-209,6$ bei 272 Fällen. Ein $\Updelta < -20$ enthält im Vergleich der CT zudem die HD I63.3 mit $-41$ ${\Delta CT}_{\text{Neuro}}^{\mathrm{UKL}}$ bei 31 Fällen.

## Diskussion

Das Fallpauschalensystem stellt Krankenhäuser vor große Herausforderungen. Die aufwandsgerechte Finanzierung der Betriebskosten durch die Krankenkassen stellt ein vielseitig reglementiertes und seit Ausgliederung der Pflegekosten in der Komplexität weiter gewachsenes System dar [1]. Der anhaltende Investitionsstau erhöht den wirtschaftlichen Druck. Die chronische Unterfinanzierung der Krankenhäuser durch die Länder zwingt die Einrichtungen zur kontinuierlichen Effizienzsteigerung und Erlössicherung, um mit Investitionen aus Eigenmitteln ihren Bestand sichern zu können [12]. Die zunehmenden Qualitäts- und Strukturvorgaben wie Mindestmengen oder Pflegepersonaluntergrenzen tun ihr Übriges. So beschrieben 45 % der im Krankenhaus-Barometer gefragten Kliniken im Jahr 2020 ihre derzeitige wirtschaftliche Situation als „eher unbefriedigend“ [11]. Abgegeben wurde diese negative Einschätzung von Häusern jeder Bettengrößenklasse. Besonders negativ sehen der Befragung nach jedoch die großen Kliniken mit mehr als 600 Betten in die Zukunft [11]. Um der geringen Steigerung des Basisfallwertes, der Pflegeausgliederung, den gedeckelten Budgets und den stockenden Investitionen seitens der Länder zu begegnen, ist es notwendig, Einsparpotenziale aufzudecken. Vor diesem Hintergrund sind vergleichende Untersuchungen mit anderen Leistungserbringern notwendig, um die eigene Situation bewerten zu können und mögliche Einsparpotenziale aufzudecken.

Radiologische Leistungen gehören neben der Nuklearmedizin zu den kostenintensivsten diagnostischen Auftragsleistungen für stationäre Fälle. Die Großgerätediagnostik ist personalintensiv und geht mit erheblichen Investitionen und Unterhaltskosten einher. Ob radiologische Diagnostik überproportional häufig angefordert wird, ist innerhalb eines Krankenhauses schwer zu bestimmen, da sie vom (internen) Zuweiser als notwendig erachtet wird, die rechtfertigende Indikation aber von der Radiologie bestätigt wird. Um festzustellen, ob hier dennoch Einsparpotenzial besteht, ist der interinstitutionelle Vergleich im Rahmen eines Benchmarks ein möglicher Weg. Der Vergleich eröffnet unter Wahrung der geltenden Finanzierungsregelungen die Identifikation von Handlungsoptionen, wie z. B. durch Verlagerung in den ambulanten Sektor. Die breiteste Datengrundlage bieten hier die Leistungsdaten des InEK. Diese Kalkulationsdaten werden regelmäßig für Benchmarks herangezogen [3, 7, 8]. Wir entschieden uns zur Limitation des Benchmarks auf die CT- und MRT-Diagnostik. Diese sind gegenüber anderen radiologischen Leistungen wie der Sonographie und der konventionellen Röntgenuntersuchung als OPS-Kode erfassbar und vom InEK für jede Fallpauschale in ihrer Anzahl benannt. Die Schnittbilddiagnostik hat eine indirekte Wirkung auf die Wirtschaftlichkeit eines Krankenhauses, ohne standardmäßig durch bestehende Kennzahlen des Berichtswesens erfasst zu werden. Während eine höhere Zahl an Untersuchungen Wartelisten, Betriebskosten und Verweildauern steigen lassen können, bleibt deren Refinanzierung über die Fallpauschalen unverändert. Ob ein Patient im Durchschnitt 1,08 MRT erhält, wie es das InEK beispielsweise in der DRG B70B für 2021 kalkuliert, oder die Untersuchung 4‑mal innerhalb des Aufenthalts durchläuft, ändert nichts an der gruppierten DRG und dem daraus resultierenden Erlös. Somit steigen die Kosten einer Behandlung, während der Erlösteil fix bleibt. Die Identifikation von hausindividuellen Behandlungspfaden, welche sich mit den Durchschnittswerten aus der InEK-Kalkulation nicht decken, kann wertvolle Anstöße zur Verbesserung der Wirtschaftlichkeit innerhalb des limitierten Vergütungssystems bieten. Nichtsdestotrotz ist jede medizinische Indikationsstellung primär im Sinne der Patientenversorgung zu prüfen.

Eine mögliche Erklärung für den erhöhten Einsatz der MRT kann u. a. der eingangs genannte erhöhte Qualitätsanspruch sowie die allzeitliche Verfügbarkeit sein. Im Beispiel des akuten ischämischen Schlaganfalls sind zudem die Indikationen für eine CT- oder MRT-Diagnostik oft in der Einzelfallentscheidung komplexer zu betrachten. So gehen die Anforderungen an die initiale Bildgebung für einen frühestmöglichen Therapiebeginn heutzutage über den reinen Blutungsausschluss mittels CT hinaus. Der Leitlinie [6] nach ist bei einer eindeutigen Symptomatik nach erfolgtem Blutungsausschluss mittels CT die Indikation einer endovaskulären Therapie mittels CT-Angiographie zu prüfen. Bei einem Symptombeginn über 4,5 h, einem unklaren Zeitpunkt des Symptombeginns oder dem Vorliegen differenzialdiagnostisch anderer Ursachen sollte eine erweitere Bilddiagnostik herangezogen werden. Hier ist die MRT gegenüber der CT besser geeignet, kleinere ischämische Hirnläsionen sehr früh zu erkennen. Darüber hinaus zeigt sie Hirnstamm-Ischämien oder mikroangiopathische subkortikale Infarkte mit größerer Sicherheit als die CT [10]. Auch können akademische Aspekte und Forschungsinteressen universitärer Einrichtungen eine weitere Erklärung für die Überschreitung der vom InEK berechneten Durchschnittswert sein.

Die vorgestellte Methodik sowie die ausgewiesenen Ergebnisse des Benchmarks zeigen, dass ein Vergleich der klinikeigenen Leistungsdaten auf Basis der Leistungszahlen der InEK-Kalkulation möglich ist. Der Benchmark weist durch den hohen Erhebungsstandard des InEK [5] sowie reliabel gebildete Vergleichskennzahlen eine hohe Datenqualität auf. Er stellt eine im Gesundheitssystem einmalige Datenbasis dar, weitgehend frei von Risiken der Datenmanipulation des Benchmark-Partners oder finanziellen Aufwendungen in der Datenerhebung [4]. Dabei ist die Validität des InEK-Benchmarks prinzipiell auch für Universitätskliniken mit besonders aufwändigen Leistungen gegeben. Aufgrund der Berücksichtigung der Leistungsdaten aller Versorgungsstufen ist die vorgestellte Methodik vom Grund- bis hin zum Maximalversorger anwendbar.

Gegenüber den Kostendaten des InEK ermöglichen die Leistungsdaten eine einheitliche Vergleichsbasis ohne einen Einfluss von klinikindividuellen Verteilungsschlüsseln. Mittels der einheitlichen Termini der DRG-Fallpauschalen, ICD-Ziffern sowie OPS-Schlüssel können valide Vergleichskennzahlen gebildet werden, welche einen Benchmark bis auf die Ebene der Fachabteilung und Hauptdiagnosen zulassen. In Zusammenhang mit dem vorgestellten Benchmark auf Basis der jährlichen Kalkulationsdaten kann die Schnittbilddiagnostik auch im Zeitverlauf betrachtet und anhand der vorgestellten Kennzahlen überwacht werden. Die Zuordnung der Leistungen und somit der Abweichungen nach entlassender Fachabteilung ist dabei durchaus zulässig, da 89,7 % der Patienten innerhalb eines Krankenhausaufenthalts einzig in der entlassenden Fachabteilung versorgt werden [13]. Außerdem ist durch diese Herangehensweise nebensächlich, ob beispielsweise eine Schädel-CT noch in der Notaufnahme, oder anschließend in der Neurologie erfolgt, sofern der Patient dort entlassen wird.

Hinsichtlich der Ergebnisbewertung ist die Kooperation von Betriebswirten und Medizinern wichtig, um möglichst alle auf den Fachbereich wirkenden Faktoren berücksichtigen zu können und die Erfolgsaussichten der zu definierenden Maßnahmen zu erhöhen. Ein funktionierendes Netzwerk, Kooperationen und ein sicher geplantes Management sind uneingeschränkte Voraussetzungen für einen effizienten Einsatz der mitunter begrenzten radiologischen Ressourcen. Die Frage nach der Notwendigkeit einer – insbesondere stationär durchgeführten – diagnostischen Prozedur führt immer wieder zu Diskussionen zwischen Leistungserbringern und Kostenträgern. Analog zum InEK-Benchmark auf Kostendatenbasis [3, 8] dienen die Kennzahlen des Leistungsbenchmarks nicht zur Aufnahme in einem automatisierten Berichtswesen und stellen keine pauschalen Sollwerte für die Leistungserbringung dar. Die Überschreitung der InEK-Kalkulationsdaten können beispielsweise sowohl auf eine überdurchschnittlich hohe Qualität der Patientenversorgung, auf ineffiziente Prozesse oder eine Unterversorgung der Radiologie im ambulanten Sektor zurückzuführen sein. Um die Einflussfaktoren der Überschreitungen auszumachen, können die Kennzahlen durch weitere externe Benchmark‑, Prozess- oder auch Einweiseranalysen erweitert werden. Die Analysen können die Ergebnisse des InEK-Benchmarks validieren und dabei helfen, konkrete Ansatzpunkte für geeignete Maßnahmen zu definieren. Sie stellen eine Orientierungshilfe und keinesfalls eine verpflichtende Grundlage für die Anforderung einer bildgebenden Diagnostik dar. Letztendlich bleibt jede Indikationsprüfung eine individuelle Entscheidung in der Patientenversorgung.

Methodenkritisch ist die Aktualität der Datenbasis zu nennen. Die Diskussion und Erarbeitung von Verbesserungsmaßnahmen erfordert stets eine Prüfung hinsichtlich der zwischenzeitlichen Veränderungen. Innerhalb der 2 Jahre der sog. *Kalkulationslücke* können Fachbereiche umstrukturiert, geschlossen oder neu eröffnet werden. Diese Lücke entsteht durch Heranziehen der Kosten- und Leistungsdaten von 2019 für das DRG-System 2021. Zudem wird das Ziel des Benchmarkings, *vom Besten zu lernen*, durch das InEK-Benchmarking nur bedingt erfüllt, da die Kalkulationsdaten ausschließlich das *Lernen vom Durchschnitt* ermöglicht. Limitationen bestehen auch in den interinstitutionellen Unterschieden der Kostenstellenzuordnung, der Schwerpunktgebiete einzelner Fachabteilungen und der Leistungserfassung in der Radiologie. Schlussfolgerungen bezüglich einer Regulation des Anforderungsverhaltens muss also immer eine Detailprüfung vorangestellt werden, um eine Fehlregulation zu vermeiden. Zur Lösung des Problems sind letztlich Strukturoptimierungen wie die Möglichkeit zur Ambulantisierung und reibungslose Versorgungsstrukturen für die Radiologie (z. B. verlässliche Terminierung und Patiententransport) wirkungsvolle Maßnahmen, um Kapazitäten im Bestand freizusetzen und darüber hinaus auch stationäre Verweildauern zu adressieren. Weiterführend ist unserer Meinung nach die Verknappung der radiologischen Ressourcen der falsche Weg zur Regulierung des Anforderungsverhaltens. Vielmehr muss auf Einweiserseite ein Anreiz zum sparsamen Einsatz der Diagnostik geschaffen werden, z. B. einer leistungsgerechten internen Leistungsverrechnung, wobei aber immer der Qualitäts- und Sicherheitsstandard für die Patienten im Blick gehalten werden muss.

Wenngleich die vorgestellte Methodik mit weiteren Analysen vergesellschaftet sein sollte, bildet sie innerhalb der viel diskutierten dualen Krankenhausfinanzierung eine objektive Grundlage zum Diskurs. Der Benchmark kann für jedes Krankenhaus erstellt werden und bildet mit den innerhalb eines Jahres erbrachten und an das InEK gemeldeten Leistungen ein realistisches Bild der Behandlungspfade einer DRG ab. Die Möglichkeit der Konstatierung des $\Updelta$ bis auf die Ebene der Hauptdiagnosen ermöglicht zudem einen Perspektivwechsel: weg vom problemorientierten Denken der hohen Auslastung innerhalb der Schnittbilddiagnostik, hin zu einem lösungsorientierten Ansatz mit dem Ziel der bestmöglichen Kapazitätsnutzung und Weiterentwicklung.

## Fazit für die Praxis

- Der InEK-Benchmark bildet eine wertvolle Ergänzung bisheriger Krankenhausmanagementtools zur wirtschaftlichen Steuerung der personellen und materiellen Ressourcen.
- Voraussetzung für den Erfolg des Benchmarks ist die Validierung der Ergebnisse durch das Controlling im Schulterschluss mit der Klinik, um diese in den klinischen Kontext zu übersetzen.
- Der Mehrwert, welcher abschließend für das UKL (Universitätsklinikum Leipzig) durch die vorgestellte Methodik verdeutlicht werden kann, ist auf jedes Krankenhaus mit einer Abrechnung nach dem aG-DRG-System zu übertragen.
- Der InEK-Benchmark auf Basis von Leistungsdaten kann Kliniken somit wichtige Impulse zur Kosteneinsparung innerhalb des reglementierten Vergütungssystems geben, wobei die Ergebnisse immer erst differenziert betrachtet werden müssen, um eine vorschnelle und qualitätsschädigende Überregulation der medizinisch notwendigen Diagnostik zu vermeiden.
